# Prospective, Double‐Blinded, Randomized Study of Topical Almond Oil and Tretinoin on the Appearance of Facial Wrinkles

**DOI:** 10.1111/jocd.71279

**Published:** 2026-11-09

**Authors:** Zill‐e‐huma Khan, Andy Lee, Alejandra Aguilar, Sarah Adnan, Nasima Afzal, Raja K. Sivamani

**Affiliations:** ^1^ Integrative Research Institute Sacramento California USA; ^2^ South Texas Health Systems McAllen McAllen Texas USA; ^3^ Texas A&M College of Medicine Bryan Texas USA; ^4^ College of Medicine, California Northstate University Elk Grove California USA; ^5^ Integrative Skin Science and Research Sacramento California USA; ^6^ Pacific Skin Institute Sacramento California USA; ^7^ University of California, Davis School of Medicine Sacramento California USA

**Keywords:** almonds, pigmentation, skin aging, tocopherol, tretinoin, wrinkles

## Abstract

**Background:**

Retinoids are widely used in the treatment of wrinkles and photoaging. However, they frequently cause irritation and increase sun sensitivity. As a result, there has been greater emphasis for the evaluation of alternative ingredients and naturally derived ingredients. Almond oil, rich in unsaturated fatty acids and antioxidant vitamin E, has been used traditionally for facial care and anti‐aging regimens. However, there have not been any comparative studies on the use of topical tretinoin on its impact on facial wrinkles.

**Aims:**

To evaluate the effects of topical almond oil (with and without vitamin E) versus tretinoin 0.025% on the appearance of facial wrinkles and photoaging.

**Patients/Methods:**

In a 16‐week randomized, double‐blind trial, 79 participants were assigned to daily facial application of either almond oil, almond oil + 0.5% vitamin E, or tretinoin 0.025%. Image‐based quantification of wrinkles, skin hydration, and pigmentation was assessed at baseline and Weeks 4, 8, 12, and 16.

**Results:**

By Week 16, all groups achieved a significant reduction in the appearance of wrinkle severity (*p* < 0.001) with no between‐group differences. The almond oil + vitamin E group provided no additional wrinkle benefit over almond oil alone. Melanin levels significantly increased in the almond oil group at week 16 (4%, *p* = 1.8E‐6), whereas the almond oil + vitamin E group showed no significant increases.

**Conclusions:**

Topical almond oil produced wrinkle severity improvements comparable to tretinoin 0.025% by 16 weeks, supporting its use as a natural alternative to retinoids for anti‐aging. Adding vitamin E with almond oil demonstrated no significant wrinkle benefit but may help attenuate the slight pigmentation increase observed with almond oil alone. Almond oil alone appeared to naturally increase the appearance of skin pigmentation that may warrant further study in other clinical settings.

## 1 Introduction

Retinoids are widely used in many anti‐aging skincare regimens and serve as one of the gold standard ingredients for improving both wrinkles and pigmentation [1]. Many studies have shown their actions on the skin include promotion of keratinocyte proliferation, stabilization of the skin barrier, stimulation of collagen in the superficial dermis, and inhibition of metalloproteinase‐based collagen degradation [2, 3]. Despite all of these actions, one of the challenges with the use of retinoids is that they are not well tolerated. Many of the early generation retinoids that are available without a prescription cause irritation, redness, scaling, photosensitization, and hyperpigmentation in those who develop retinoid dermatitis in darker tones [4].

As a result, there is much interest in finding alternatives to retinoids that can still maintain some of the anti‐aging benefits. Ingredients such as bakuchiol and acetyl zingerone have emerged as potential alternatives, along with 3rd and 4th generation retinoids such as adapalene and trifarotene [5, 6, 7, 8, 9].

Among plant‐derived ingredients, almonds have been gaining attention due to their varied composition of compounds [10]. Almonds contain a mix of multiple fatty acids, β‐sitosterol, squalene, and α‐tocopherol (vitamin E) [11]. Vitamin E is the most abundant antioxidant in almonds and has been evaluated in both systemic and topical contexts for its dermatologic effects. However, it is more challenging to use in topical formulations because of its high content of unsaturated fatty acids, such as oleic and linoleic acids. Almond oil is susceptible to lipid peroxidation, particularly when exposed to prolonged UV radiation and heat. Lipophilic antioxidants such as α‐tocopherol have been shown to significantly inhibit oxidative degradation in oil‐based systems, including emulsions and bulk vegetable oils [12].

Despite the widespread commercial interest and use of almond oil in cosmetic formulations, there is little research on the effects of almond oil directly on the skin for its effects on photoaging endpoints such as skin hydration, wrinkle severity, and pigmentation. We previously demonstrated that daily consumption of almonds over 16 weeks demonstrated a statistically significant 8.41% reduction in wrinkle severity among postmenopausal women who received the almond intervention [13]. However, it remains unknown how almonds would impact the skin if they are delivered topically.

We conducted a randomized controlled study that aimed to compare the cosmetic effects of topical almond oil, with or without vitamin E, and tretinoin oil on the appearance of wrinkles and photoaging.

## 2 Materials and Methods

### 2.1 Study Participants

This study was a 16‐week, randomized, double‐blind, single‐center clinical trial conducted in Sacramento, California, evaluating the effects of topical almond oil on signs of facial aging in postmenopausal women. The protocol was approved by an Institutional Review Board (IRB) committee and registered on ClinicalTrials.gov (NCT# NCT06571721).

The inclusion criteria included postmenopausal women with Fitzpatrick Skin Types I, II, or III and facial fine lines or mild‐to‐moderate wrinkles. Participants were excluded if they were pregnant or breastfeeding, prisoners, adults unable to provide informed consent, individuals with known allergies to nuts or tocopherols, current or former smokers who had either smoked in the past year or a smoking history greater than 10 pack‐years, participants with an autoimmune photosensitive condition or a known genetic condition characterized by collagen deficiency, such as Ehlers–Danlos syndrome, and individuals who had undergone cosmetic facial procedures within the past 3 months or deeper chemical peels (e.g., trichloroacetic acid–based peels) within the year prior to enrollment.

Subjects were required to discontinue vitamin E‐containing supplements and dietary sources, including all nuts, sunflower seeds, and sunflower oil, as well as topical cosmetic products known to modulate collagen or pigmentation during both a two‐week washout period and the duration of the study. Products requiring washout included topical retinoids (such as tretinoin, adapalene, or retinol), antioxidant ingredients such as vitamin C or vitamin E, and pigment‐reducing agents such as hydroquinone, azelaic acid, and kojic acid. Participants agreed not to use any topical products containing nut oils or nut extracts during the study period.

### 2.2 Study Design and Intervention

A total of 90 participants were enrolled in the study. 79 participants in total successfully completed all visits. All participants provided written informed consent before enrollment. Subjects were randomized equally (1:1:1 allocation, 30 per group) to receive one of three treatments for the study duration.

### 2.3 Wrinkle Severity

High‐resolution 3D photographs were taken at baseline and Weeks 4, 8, 12, and 16. Images were analyzed using digital surface topography software to generate quantitative wrinkle severity scores. Participants acclimated for 15 min in a temperature and humidity‐controlled room prior to imaging.

### 2.4 Skin Hydration

Cheek hydration was assessed bilaterally using a corneometry‐based device (MoistureMeter SC, Delfin Technologies). Readings were taken in duplicate from each cheek and then averaged. Measurements were performed at baseline and all follow‐up visits.

### 2.5 Transepidermal Water Loss (TEWL)

TEWL was measured on each cheek using a Vapometer device (Delfin Technologies) to assess epidermal barrier function. Duplicate readings were averaged per cheek and across both cheeks.

### 2.6 Sebum Excretion Rate

Sebum production was measured on both cheeks using Sebumeter tape strips (Courage & Khazaka SM815). Readings were taken in duplicate per cheek and averaged. Sebum excretion rate was reported as micrograms of sebum per square centimeter.

### 2.7 Pigmentation and Erythema

Melanin index and erythema index were measured using a SkinColorCatch colorimeter. This device provided quantitative values for pigmentation and redness at each time point. Measurements were taken in duplicate per cheek and averaged.

### 2.8 Subjective Tolerability

Participants completed a validated tolerability questionnaire at Weeks 4, 8, 12, and 16. They rated symptoms including itching, burning, stinging, redness, and pigment changes on a 0 (none) to 3 (severe) scale.

### 2.9 Statistical Analysis

All data are reported as mean ± standard error of the mean (SEM) unless otherwise noted. Results were analyzed using a paired Student's *t*‐tests where a *p*‐value < 0.05 was deemed statistically significant. Between‐group comparisons were assessed using one‐way ANOVA. When significant, post hoc pairwise comparisons were performed using Tukey's correction for multiple comparisons. A *p*‐value < 0.05 was considered statistically significant. A Chi‐squared analysis was performed on categorical data where *p* < 0.1 was considered statistically significant. Due to the exploratory nature of this randomized controlled trial as well as limited sample size, *p* < 0.1 was prespecified to minimize Type II error. Significances for all graphs with the following: * = *p* < 0.05, ** = *p* < 0.01, *** = *p* < 0.005. Only data from enrolled subjects that received the study intervention were analyzed.

## 3 Results

### 3.1 Subjects

A total of 90 participants enrolled in the study, of whom 79 completed all study visits (Figure 1). There were 26 participants in the Almond Oil + Vitamin E group (62.1 ± 1.39 years), 25 in the Tretinoin Oil + Castor Oil group (60.3 ± 1.48 years), and 28 in the Almond Oil only group (59.5 ± 1.20 years). Fitzpatrick skin Types I–III were represented across all groups.

**FIGURE 1:**
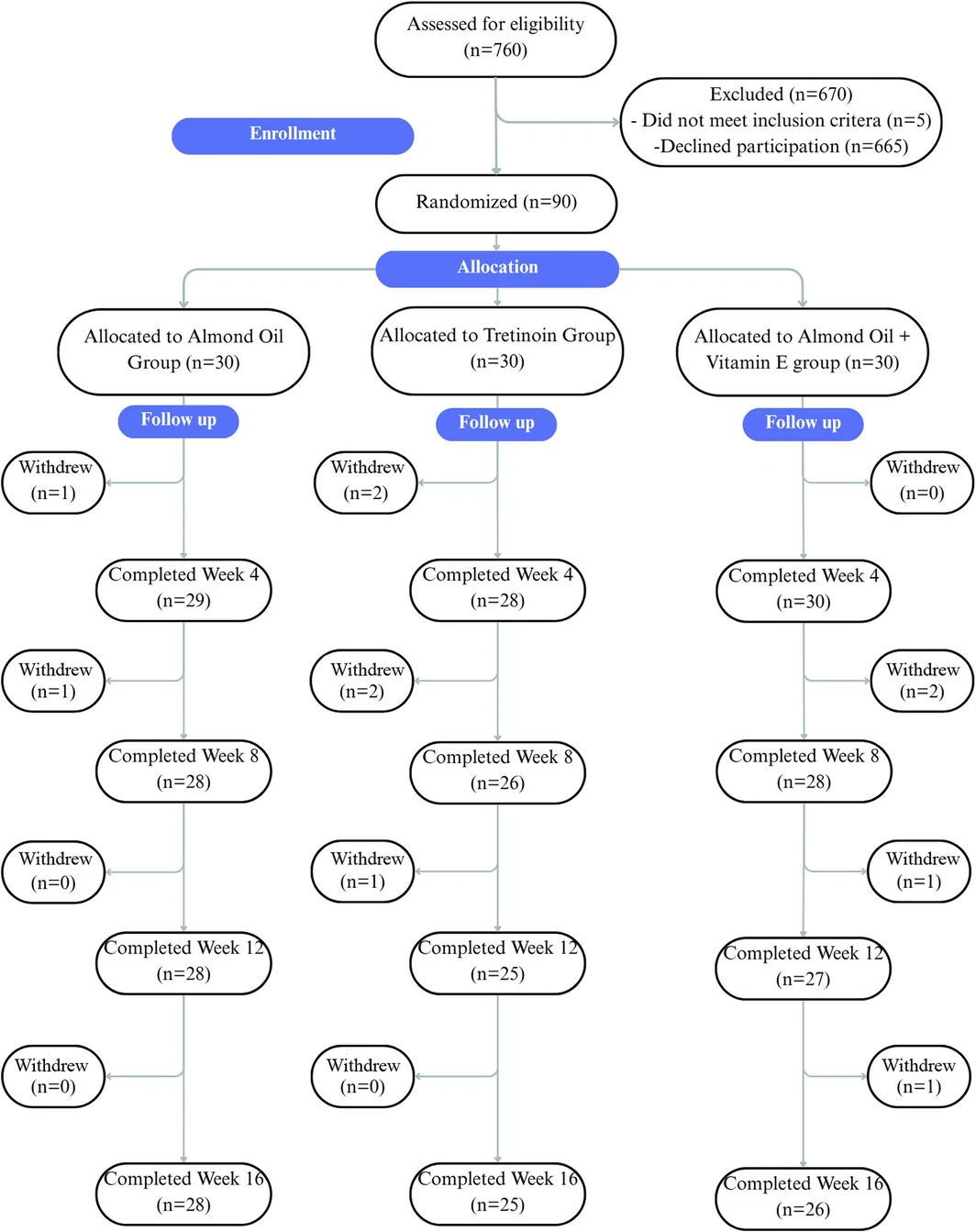
Consolidated standards of reporting trials (CONSORT) diagram.

### 3.2 Wrinkle Severity

At 4 weeks, none of the groups demonstrated statistically significant changes in wrinkle severity when compared to baseline (Figure 2). The Almond Oil + Vitamin E group showed a significant 2% decrease at Week 8 compared to baseline (*p* = 0.032). There was a significant decrease at Week 12 (3%, *p* = 0.0019) and at Week 16 (4%, *p* < 5E‐4) when compared to baseline. The Tretinoin group showed a similar finding of a significant decrease at Week 8 (3%, *p* = 0.001), Week 12 (3%, *p* = 0.0031) and Week 16 (4%, *p* = 0.0007) when compared to baseline. The Almond Oil group showed a decreasing trend of 1% at Week 8 compared to baseline; however, this was not statistically significant. Significant decreases for the Almond Oil group were found in Week 12 (3%, *p* = 1.7E‐5) and at Week 16 (4%, *p* = 1.1E‐5) when compared to baseline.

**FIGURE 2:**
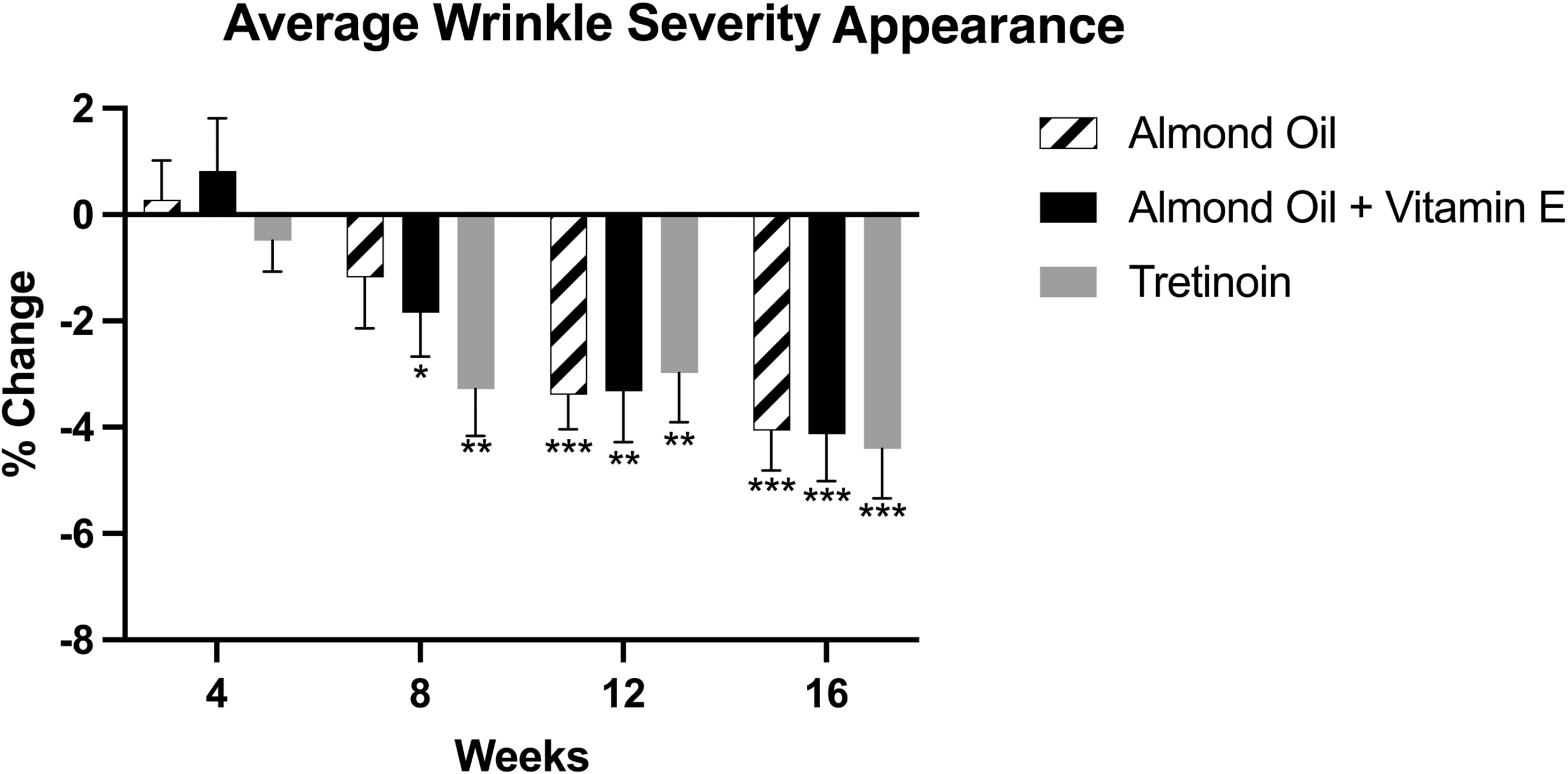
Average percent change in average wrinkle severity at Weeks 4, 8, 12, and 16 compared to baseline. Error bars represent Standard Error of the Mean (SEM). * = *p* < 0.05, ** = *p* < 0.01, *** = *p* < 0.005.

### 3.3 Skin Hydration

Skin Hydration was measured at both cheeks for all study visits (Figure 3). At Week 4, significant increases in skin hydration were observed in the Almond Oil + Vitamin E group (26%, *p* = 0.03) and the Tretinoin Oil group (32%, *p* = 0.04) compared to baseline. The Tretinoin Oil group continued to exhibit significant increases at Weeks 8 (33%, *p* = 0.03) and 12 (32%, *p* = 0.048), though these improvements did not persist through Week 16. The Almond Oil only group did not show significant changes in skin hydration until an 18% decline at Week 16 (*p* = 0.006).

**FIGURE 3:**
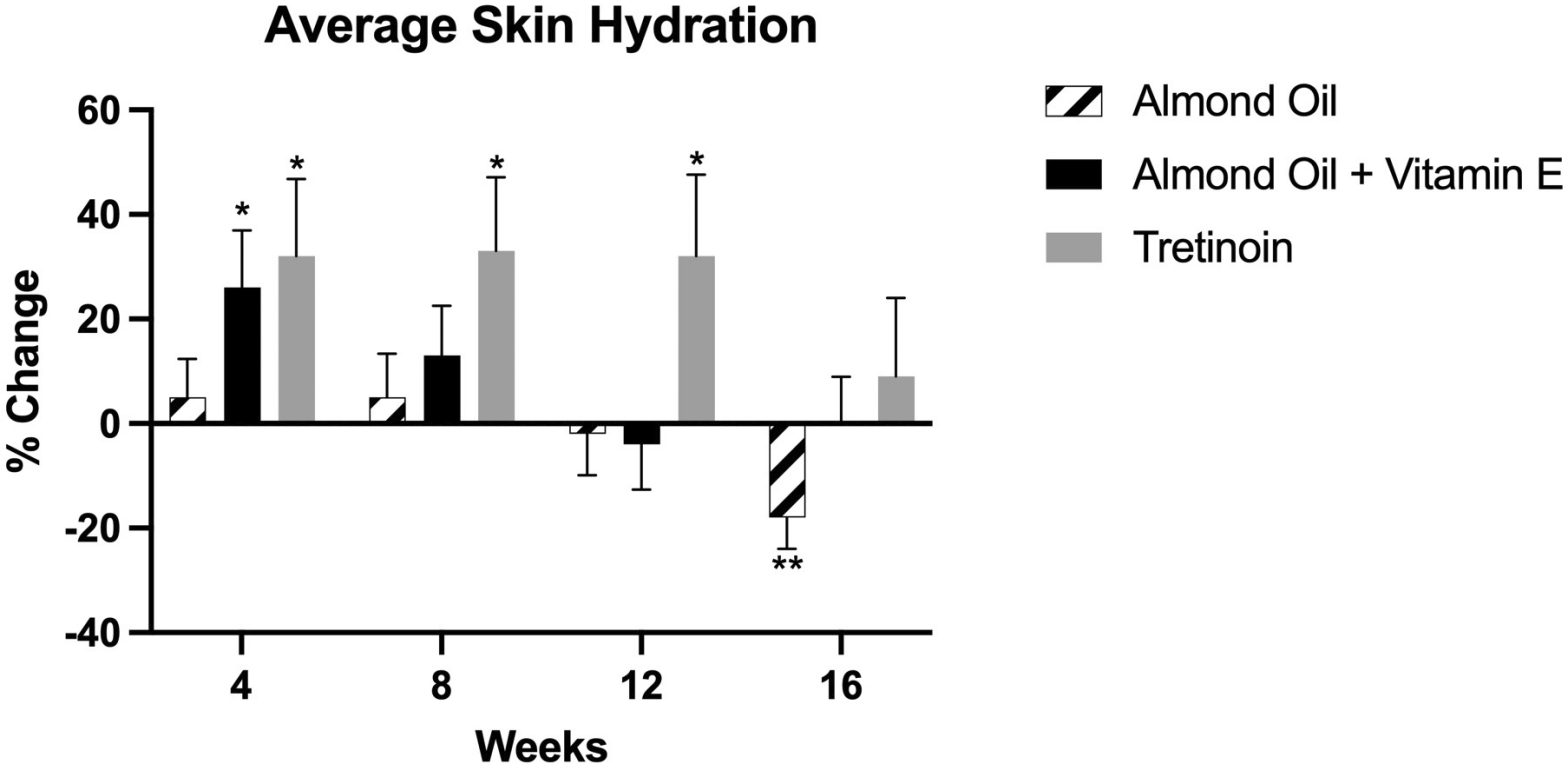
Mean percent change in skin hydration at Weeks 4, 8, 12, and 16 compared to baseline. Error bars represent SEM. * = *p* < 0.05, ** = *p* < 0.01.

### 3.4 Transepidermal Water Loss (TEWL)

Transepidermal water loss was measured at both cheeks for all study visits (Figure 4). Increases in TEWL at Week 4 compared to baseline were observed across all treatment groups: Almond Oil + Vitamin E (22%, *p* = 0.02), Almond Oil (27%, *p* = 0.02), and Tretinoin Oil (25%, *p* = 0.04). By Week 12, TEWL remained significantly elevated only in the Tretinoin Oil group (18%, *p* = 0.01). At Week 16, there were no significant changes in TEWL to baseline levels across all groups.

**FIGURE 4:**
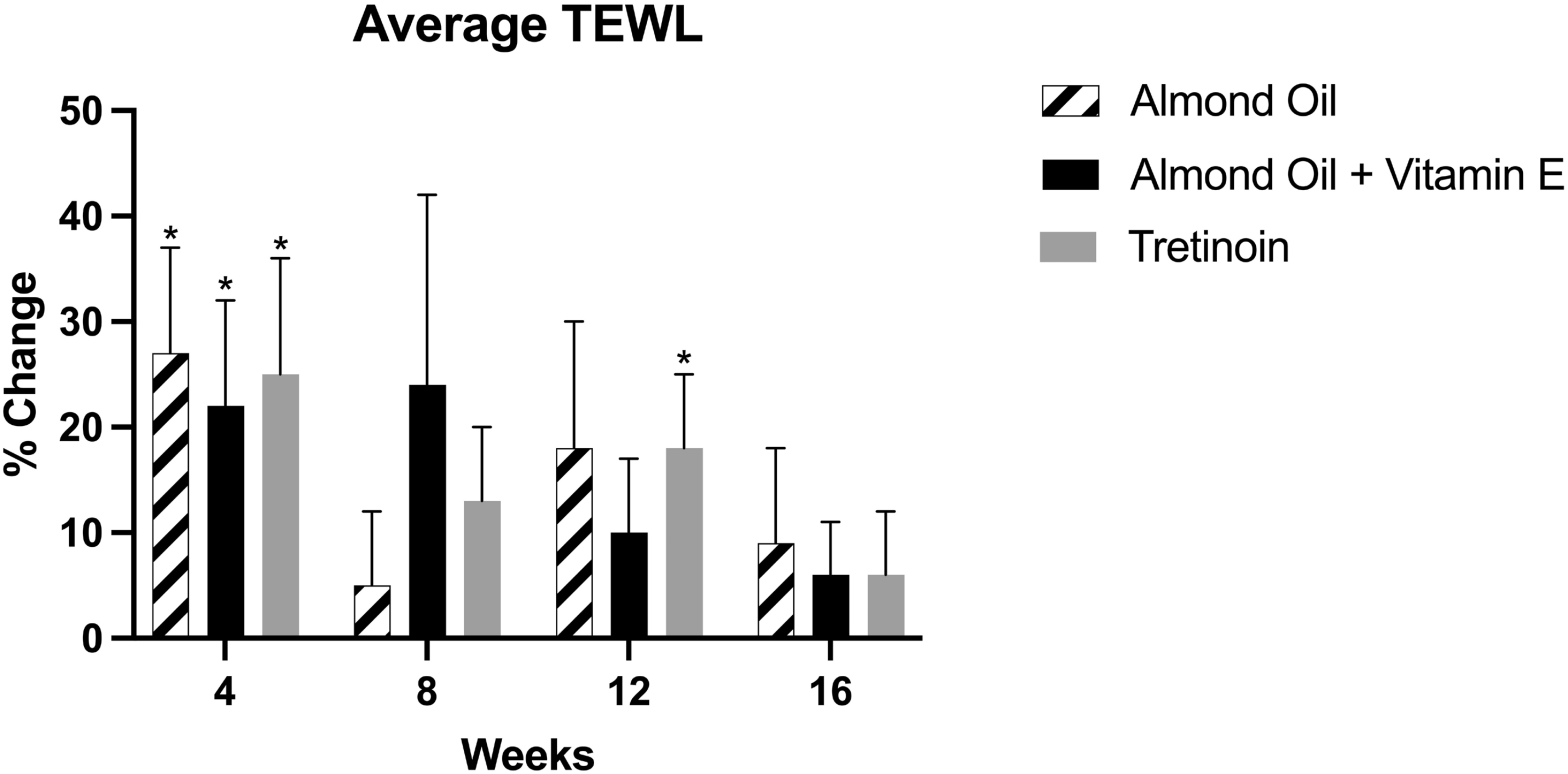
Mean percent change in TEWL at Weeks 4, 8, 12, and 16 compared to baseline. Error bars represent SEM. * = *p* < 0.05.

### 3.5 Sebum Excretion Rate

Sebum excretion was measured at both cheeks for all study visits (Figure 5). Significant increases in sebum excretion were seen in all three groups. The Almond Oil + Vitamin E group showed a significant increase at Week 12 (70%, *p* = 0.04). The Tretinoin Oil group displayed significant increases throughout the study duration in Week 4 (71%, *p* = 0.0017), Week 8 (131%, *p* = 0.038), Week 12 (160%, *p* = 0.015), and Week 16 (93%, *p* = 0.01). The Almond Oil group also displayed significant increases in sebum excretion at Week 4 (121%, *p* = 0.036), Week 8 (164%, *p* = 0.0048), Week 12 (110%, *p* = 0.0052), and Week 16 (79%, *p* = 0.03).

**FIGURE 5:**
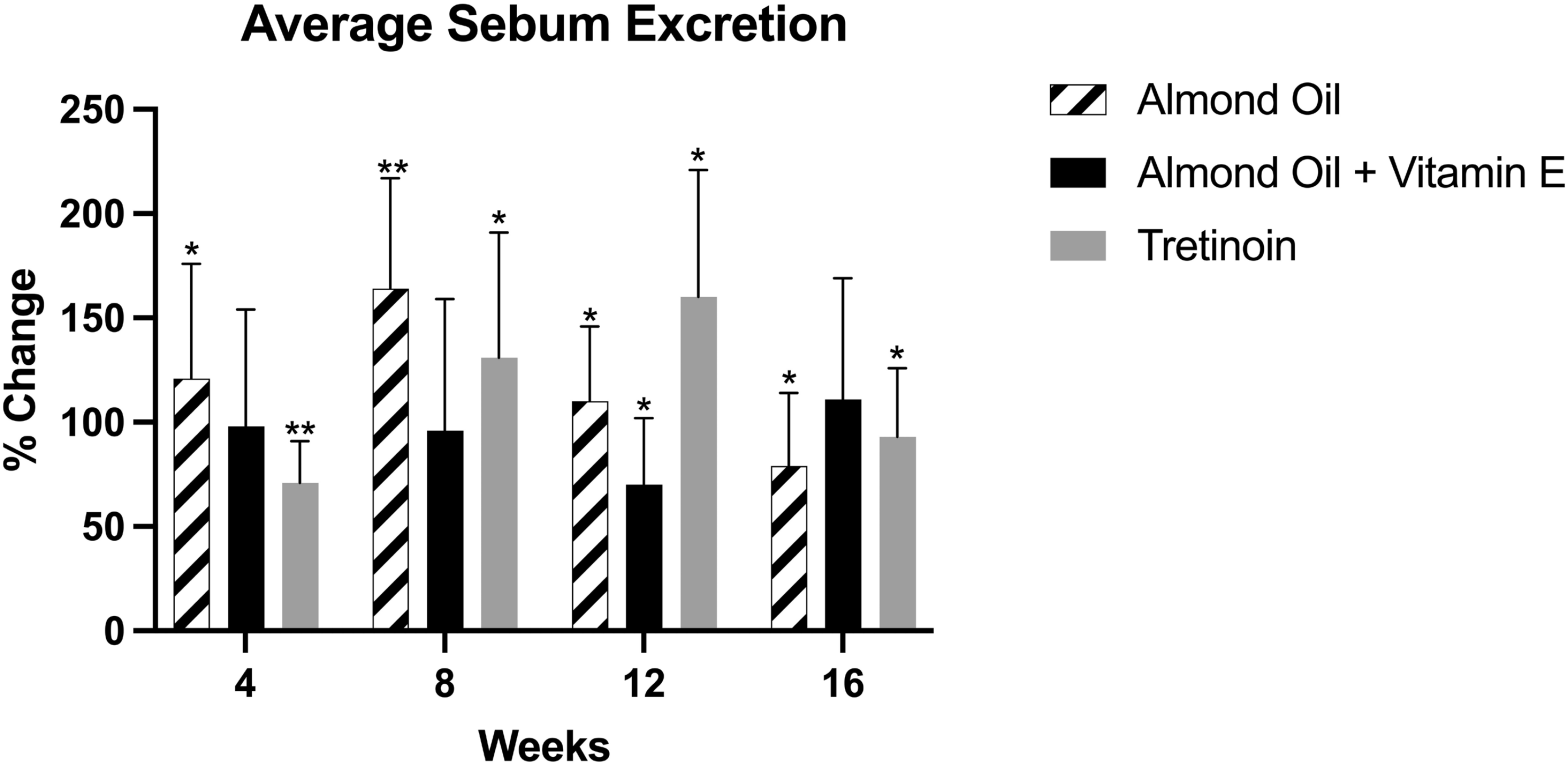
Mean percent change in sebum excretion at Weeks 4, 8, 12, and 16 compared to baseline. Error bars represent SEM. * = *p* < 0.05, **p < 0.01.

### 3.6 Erythema

Minimal changes in facial erythema were observed (Figure 6). No significant changes in erythema were noted for any treatment group across all time points when compared to baseline.

**FIGURE 6:**
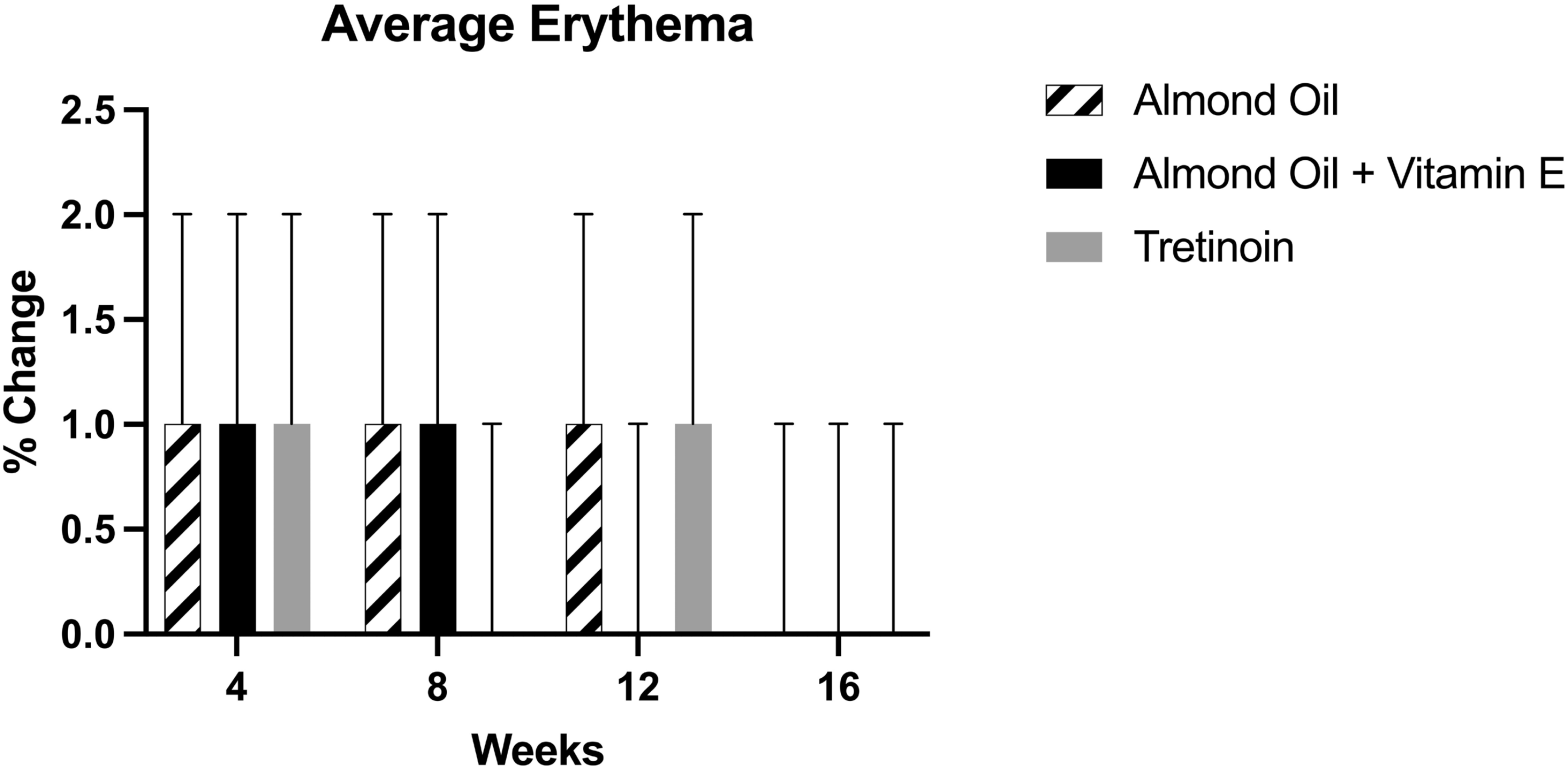
Mean percent change in erythema at Weeks 4, 8, 12, and 16 compared to baseline. Error bars represent SEM.

### 3.7 Luminosity

In the Almond Oil + Vitamin E group, no significant changes in luminosity were observed (Figure 7). At Week 4, there was a 2% decrease found but did not reach statistical significance. The Tretinoin Oil group demonstrated significant decreases at Weeks 12 (2%, *p* = 0.01) and 16 (3%, *p* = 0.025). The Almond Oil group had significant decreases starting from Week 8 (2%, *p* = 0.006), continuing through Week 12 (3%, *p* = 9E‐5) and Week 16 (5%, *p* = 0.02).

**FIGURE 7:**
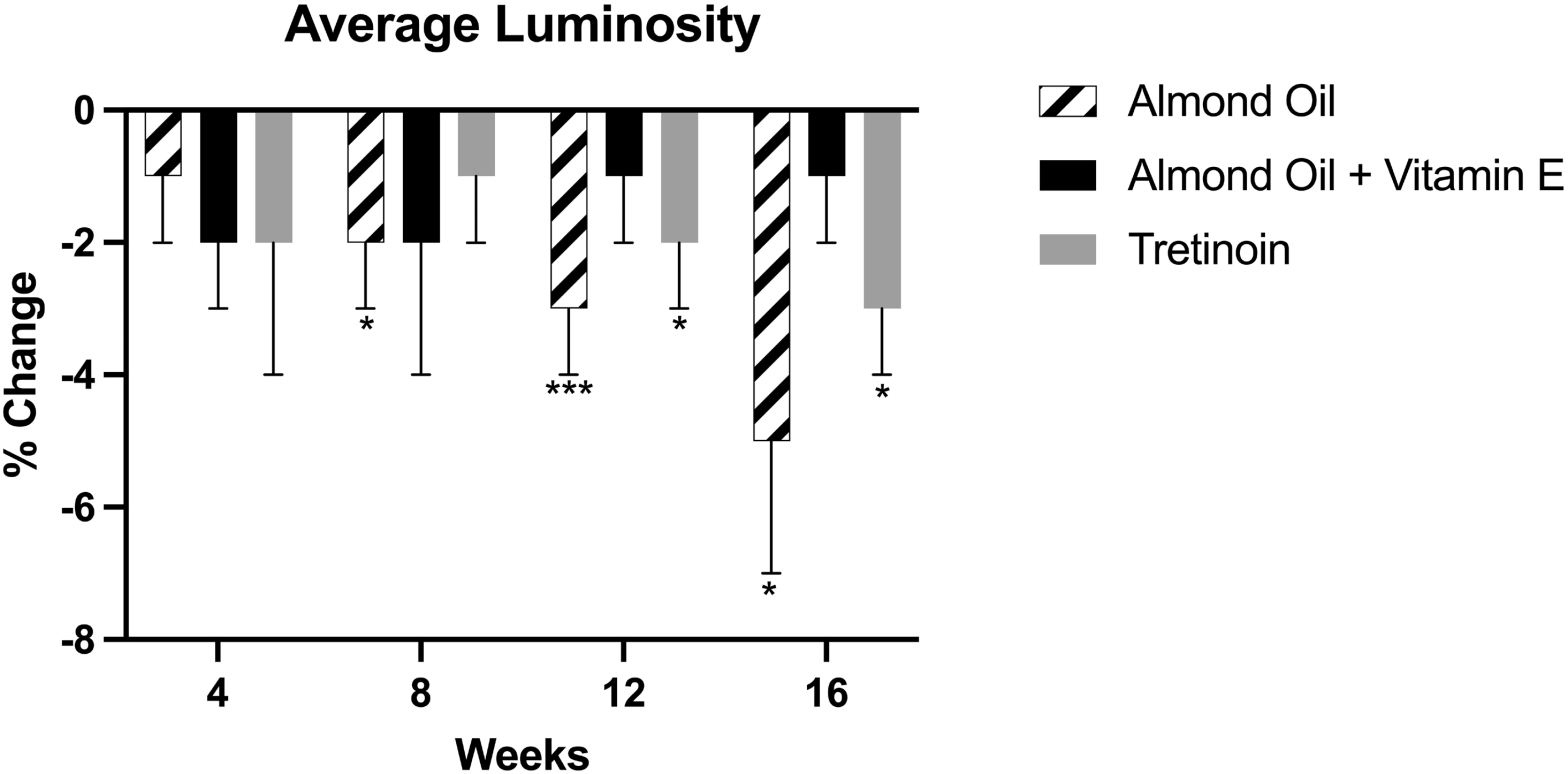
Mean percent change in luminosity at Weeks 4, 8, 12, and 16 compared to baseline. Error bars represent SEM. * = *p* < 0.05, ***p < 0.005.

### 3.8 Melanin

The Almond Oil + Vitamin E group showed no significant changes in the melanin index for all timepoints (Figure 8). A slight decreasing trend was observed (2%) but was not statistically significant. Similarly, the Tretinoin Oil group had no significant changes, though a trend toward an increase was noted at Week 12 (1%, *p* = 0.066). In contrast, the Almond Oil alone group demonstrated significant increases at Weeks 8 (1%, *p* = 0.016), 12 (3%, *p* = 0.0002), and 16 (4%, *p* = 1.8E‐6).

**FIGURE 8:**
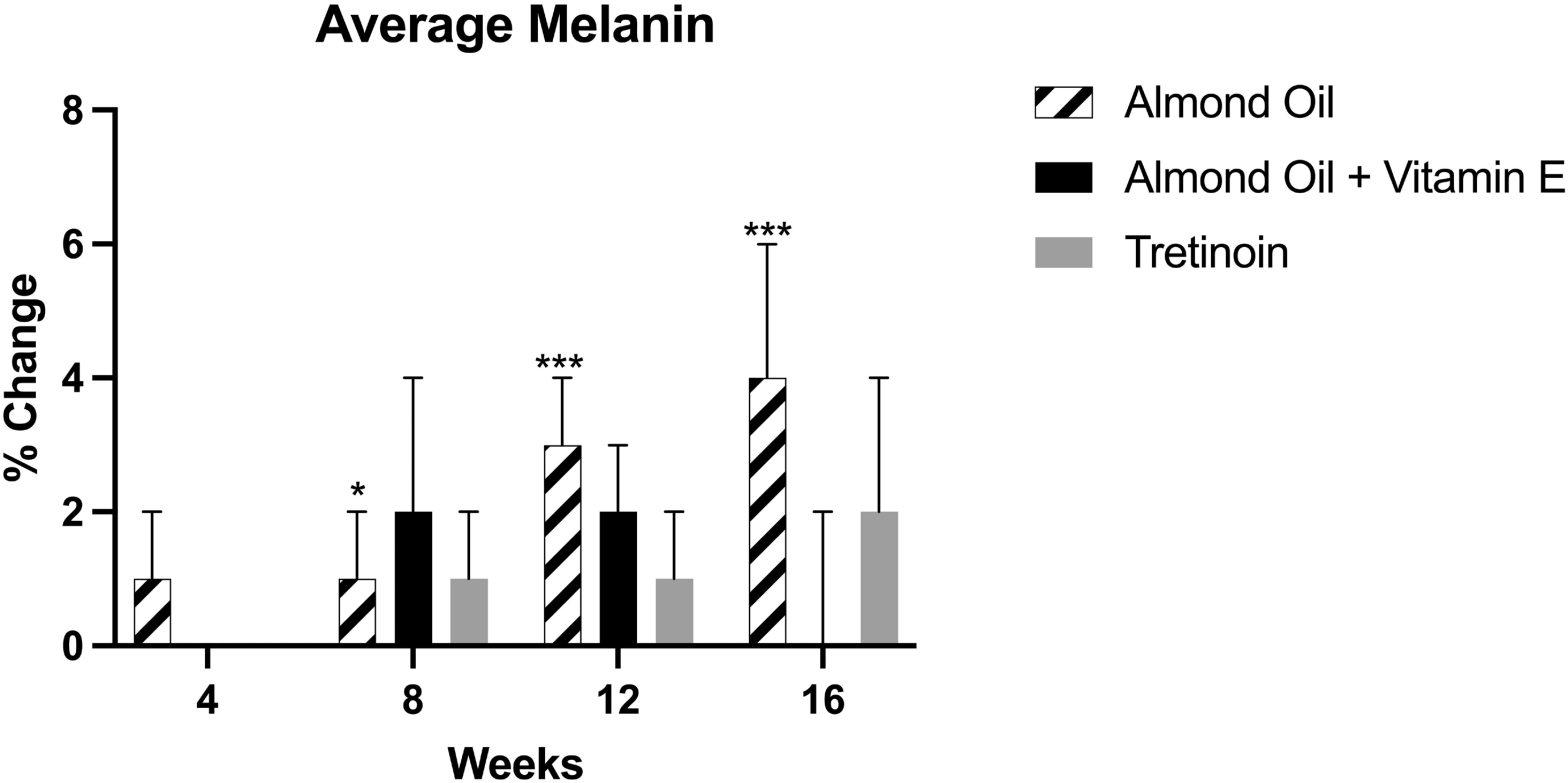
Mean percent change in melanin at Weeks 4, 8, 12, and 16 compared to baseline. Error bars represent SEM. *p < 0.05, ***p < 0.005.

### 3.9 Adverse Events

A total of 9 subjects across all treatment groups reported adverse events during the study period. In the Tretinoin Oil group, adverse effects included transient irritation, redness, and peeling (*n* = 1), bilateral erythema (*n* = 1), worsening rosacea (*n* = 1), and facial breakouts (*n* = 2), with two subjects discontinuing early due to skin reactions. One subject also reported an episode of shingles, which was deemed to not be related to the topical and resolved without intervention. In the Almond Oil + Vitamin E group, isolated cases of nose papules (*n* = 1) and perioral irritation with scaling and cracking (*n* = 1) were reported, both of which were mild and resolved. In the Almond Oil group, one subject experienced a flare of acne that persisted through the study. No serious adverse events were reported.

## 4 Discussion

Our findings suggest that daily topical application of almond oil had comparable results to tretinoin 0.025% at Week 16 in improving image‐based wrinkle severity, and the addition of vitamin E to the almond oil did not offer any additional incremental benefit. The tretinoin group appeared to work faster at reducing wrinkle severity by 8 weeks, but all the groups appeared to have a similar improvement by Week 12 and Week 16.

Sweet almond oil is naturally rich in unsaturated fatty acids (approximately 70% oleic acid and 20% linoleic acid) and contains compounds such as α‐tocopherol, squalene, and phytosterols that may add to the benefits of skin anti‐aging. A preclinical study demonstrated that topical almond oil is capable of preventing UVB‐induced structural damage and photoaging in a mouse model, highlighting its photoprotective potential [11]. Vitamin E has been shown to improve skin integrity through different mechanistic pathways, such as inhibiting protein kinase C activation and thereby reducing the expression of collagenase (MMP‐1), an enzyme responsible for collagen breakdown [14]. By inhibiting collagen degradation, vitamin E may help preserve dermal structure and elasticity. It also contains antioxidant properties that combat oxidative stress in the skin, increasing glutathione levels in keratinocytes and preventing further cellular damage that could lead to wrinkle formation. However, in this study, the Almond Oil + Vitamin E group did not exhibit significantly greater efficacy than the Almond Oil group, suggesting that almond oil already contains sufficient effect size that the addition of vitamin E did not offer additional benefit.

A consistent significant increase in sebum secretion was seen in the almond oil group and tretinoin oil group that started at Week 4, while the Vitamin E group only saw a significant increase at Week 12. Sebum helps maintain the skin's lipid barrier and prevents excessive transepidermal water loss. The reason for the increased sebum excretion rate in all groups is not clear, but sebum contributes to protecting the skin barrier and keeping the skin supple, moisturized, and maintaining elasticity, with known benefits for improving fine lines and wrinkles [15]. With age, there is sebaceous gland atrophy and decreased sebum production, which is thought to contribute to skin dryness and wrinkle formation, especially in areas of the face that contain fewer sebaceous glands [16]. Therefore, significant sebum increases may have played a role in wrinkle severity reduction.

Interestingly, our study saw a significant increase in melanin index observed exclusively in the Almond Oil group. Beginning at Week 8, participants in this group demonstrated a 1% increase in melanin levels from baseline, which further increased to 3% at Week 12% and 4% by Week 16. This progressive rise in melanin was not observed in the Tretinoin or Almond Oil + Vitamin E groups, suggesting that plain almond oil may influence melanogenesis over time. Almond oil contains a mixture of fatty acids with varying effects on pigmentation pathways. While unsaturated fatty acids such as oleic and linoleic acid have been shown to inhibit melanin synthesis, saturated fatty acids like palmitic acid may promote melanogenesis [17, 18, 19]. The absence of a significant melanin increase in the Almond Oil + Vitamin E group raises the possibility that α‐tocopherol may have attenuated this pigmentation response. Vitamin E has been shown to interfere with melanin synthesis by inhibiting tyrosinase activity and enhancing intracellular antioxidant defenses, potentially counteracting the melanogenic effect of certain lipid components in almond oil.

The increased melanin in the Almond Oil only group may have other potential clinical uses that warrant further exploration. While this study was focused on photoaging, there should be further study to assess whether the darkening effect of almond is reproduced in a larger study as an alternative to intentional tanning.

This study, to our knowledge, is the first clinical trial to directly compare topical almond oil (with or without vitamin E) to tretinoin in its anti‐aging outcomes. The results demonstrate that almond oil can produce statistically significant improvements in wrinkles and skin health that are comparable to conventional tretinoin treatment at 16 weeks, supporting its potential as a natural, evidence‐based alternative option for anti‐aging skincare. Future research should expand on these findings by involving a more diverse study population, examining longer‐term use, and exploring dual approaches (such as diet + topical) to maximize anti‐aging skin benefits of almonds.

This study has several limitations that should be considered. First, this was a three‐arm active‐comparator study without a vehicle or untreated control group. However, there is no suitable control or vehicle for almond oil since the application is based on whole almond oil rather than on prespecified active ingredients within almond oil. The study was limited to postmenopausal women with Fitzpatrick skin Types I–III, limiting the generalizability of the findings to darker skin types. Also, the study was conducted over 16 weeks, and longer‐term changes to the skin beyond 16 weeks of application are not known. Tretinoin is known to impact other changes in the skin, such as stimulation of collagen and reduction of the activity of MMP‐1. These actions were not studied here, and the effect of almond oil on the superficial dermis enzymes is unknown. Finally, future studies should include an expanded population of women and include men as well. A longer‐duration study should be considered in the future based on the results of this study.

## Author Contributions

Conceptualization, R.K.S.; Methodology, R.K.S., N.A.; Formal Analysis Z.K., A.L., N.A., R.K.S.; Investigation, Z.K., A.A., S.A.; Data Curation, Z.K., A.L., S.A., N.A.; Writing – Original Draft Preparation, Z.K., A.L.; Writing – Review and Editing, A.L., N.A., R.K.S.; Visualization, Z.K., A.L.; Supervision, R.K.S.; Project Administration, N.A., R.K.S.; Funding Acquisition, R.K.S. All authors have read and agreed to the published version of the manuscript.

## Funding

This research was funded by The Almond Board of California (Modesto, CA, USA).

## Disclosure

R.K.S. has served as a consultant or speaker for Amgen, Burt's Bees, Sanofi, Bristol Myers Squibb, Arbonne, Trace Minerals, Codex Labs, Pfizer, Phothera, Almirall, Nutraceutical Wellness, Lilly, Galderma, Incyte, Novartis, Arcutis, Janssen, Abbvie, Leo, UCB, Sun, and Regeneron Pharmaceuticals.

## Ethics Statement

The study was conducted according to the guidelines of the Declaration of Helsinki and approved by the Allendale Institutional Review Board (IRI24_01_AlmondOil) on July 19, 2024.

## Consent

Written informed consent was obtained from all subjects and/or guardians involved in the study.

## Conflicts of Interest

The authors declare no conflicts of interest.

## Data Availability

The data that support the findings of this study are available from the corresponding author upon reasonable request.
